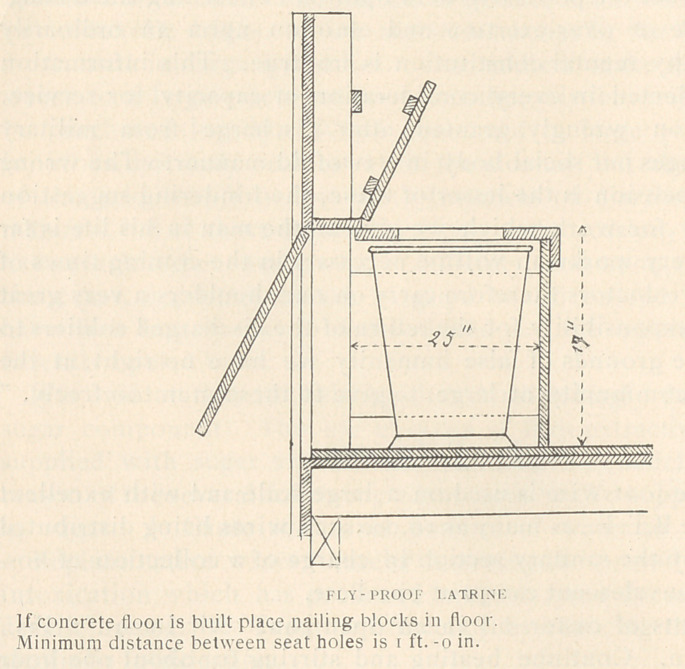# Circulars, Bulletins, and Reports Issued from the Office of the Chief Surgeon of the American Expeditionary Forces

**Published:** 1918-08

**Authors:** 


### CIRCULARS, BULLETINS AND REPORTS

ISSUED FROM THE OFFICE OF THE CHIEF SURGEON, OF THE AMERICAN EXPEDITIONARY FORCES IN FRANCE.

*Under this heading will be published extracts from circulars and bulletins issued by the Chief Surgeon of the Medical Department of the American Expeditionary Forces in France. It is believed that these will be of general interest and value to medical officers.*

### EXTRACTS FROM C. S. O. CIRCULARS

### FOOD AND NUTRITION SECTION.

Announcement is made of the organization of a Food and Nutrition Section in the Division of Sanitation, Office of the Chief Surgeon, A. E. F. This Section will be under the supervision of the Director of Laboratories and Infectious Diseases, and its functions shall be to inspect, investigate, and make recommendations concerning those factors directly affecting the nutrition of troops of the American Expeditionary Forces. The Section is authorized to advise concerning the suitability of rations and dietaries, and all changes or substitutions proposed in rations and dietaries for troops, hospitals, or prison camps; and in cooperation with the Quartermaster Department, the Section will devise and propose measures for the observation of food.

### SURGICAL OPERATIONS.

*a*) Surgical operations of election for chronic conditions which existed before the war and which do not incapacitate for the performance of ordinary duty, will not, as a rule, be performed during periods of military activity, and will only be done in well equipped base or camp hospitals of the A. E. F.

*b*) Hernias should be operated upon subject to the foregoing restrictions, bearing in mind military convenience and the extent of present or threatened disability.

*c*) Operations for varicocele should, as a rule, not be performed at all.

*d*) Removal of tonsils is not to be done, except when marked obstruction to respiration exists, or when they are a source of infection in a systemic disease.

*e*) Hemorrhoids should be operated upon subject to the restrictions of Paragraph *a*.

*f*) Special instructions for the handling of orthopedic patients are in course of preparation.

### Proper Handling and Disposition of Slightly Wounded Men.

Attention is directed to the importance of early proper handling and disposition of slightly wounded men in all hospital formations. While the handling of the seriously wounded usually entails a greater exercise of technical skill, the claims of the slightly wounded for equal attention may be overlooked. It must be borne in mind that a neglected or improperly treated slight wound may have serious consequences and cause prolonged hospitalization. Slightly wounded men form the greatest military asset among all those admitted to hospitals, in that their early return to duty can be looked for if they are properly treated. The tendency in some hospitals is to delegate the care and treatment of slightly wounded men to the medical officers young in experience and skill in surgery.

Without deflecting the full measure of attention to be given to serious cases, surgical personnel at hospitals should be so assigned as to bring skill and attention to bear upon slightly wounded men equal to that given to more serious cases, carrying into effect that principle of military surgery which contemplates the greatest good to the greatest number.

### Etiquette of Visits to French Hospitals.

Correspondence recently received from the French Service de Santé indicates that in certain cases medical officers of the A.E.F. have visited American patients in French hospitals without first calling on the Médecin Chef of the hospital to get his permission.

It is a military principle which governs in all armies, to which the French attach much importance, that an officer should not go into any military organization for the purpose of inspecting without first calling on the Commanding Officer of that organization to get his permission. It is very desirable when the visit is one of inspection, and not merely a personal visit to individual patients, that the Médecin Chef or an officer designated by him should accompany the American medical officer. This is an important matter of military administration, as well as military courtesy, which all medical officers should be careful to observe.

### Hospitalization and Evacuation of Cases of Pulmonary Tuberculosis and Suspected Pulmonary Tuberculosis.

*a*) Collecting and observation centers have been established at various hospitals for cases of pulmonary tuberculosis and suspected pulmonary tuberculosis which may occur in the A. E. F.

*b*) In future the diagnosis " Pulmonary Tuberculosis " should be limited to cases in which tubercle bacilli are found in the sputa. Cases in which this diagnosis has been established should be evacuated to Base Hospitals, which are designated as collecting centers for these cases during the period preceding their evacuation to the United States.

*c*) Cases of suspected tuberculosis should be diagnosed " Tuberculosis Observation ". Such cases should be evacuated to Base-Hospitals, which are designated for the purpose.

### Organization of Professional Services Medical Department, A. E. F.

There has been appointed, by G.O. 88, G.H.Q., A.E.F., June 6, 1918, for the Medical Department :

A Director of Professional Services, A.E.F.

A Chief Consultant, Surgical Service, A.E.F.

A Chief Consultant, Medical Service, A. E. F.

Senior Consultants in special sub-divisions of surgery and medicine.

Division Specialists, and

Consultants for base hospital centers and other formations.

In order to utilize the professional services of the Specialists of the Medical Department, A.E.F., in a manner which will best facilitate complete co-ordination between forces from front to rear, the following instructions are issued :

### Director of Professional Services.

The Director of Professional Services, under the Hospitalization Division of the Office of the Chief Surgeon, will supervise the professional activities of the Medical Department, A. E. F., and co-ordinate the work of the Consultants and Specialists of the Medical Department.

### Chief Consultants.

The Chief Consultant, Surgical Service, will supervise the professional surgical sub-divisions in the A.E.F. He will organize and co-ordinate these divisions in a manner which will permit him to anticipate, as far as possible, necessary changes in personnel so that timely request for such changes may be made. He is responsible for the proper formations of the surgical teams in the A.E.F., and those attached to the units of the Allies, and he will keep lists and records of the teams whereby the amount and the efficiency of their work may be checked. For this purpose, he will require from each surgical team suitable monthly reports of the number of operations performed and the results obtained. He will make such recommendations as he may deem necessary for inspections as to technical procedure and instruction, details of operating surgeons, details to surgical teams, and appointment of Surgical Consultants in the A. E F.

The Chief Consultant, Medical Service, will supervise all medical subdivisions in the A. E. F., and will make such recommendations as may be necessary to insure a high professional standard and complete harmony among his assistants functioning in all formations.

### Senior Consultants.

Under supervision of the Director of Professional Services and the Chief Consultants in surgery and in medicine, Senior Consultants of the special sub-divisions of medicine and surgery will co-ordinate professional activities relating to their specialities.

They will make such recommendations to the Chief Consultant as are deemed necessary for the instruction of consultants and specialists in divisional and other army formations, in order that prompt execution of directions relative to professional subjects may be assured.

### Senior Divisional Consultants.

One Senior Medical and one Senior Surgical Consultant will be assigned to all tactical organizations which are the equivalent of one Army Corps, and consultants will be appointed in such numbers as may be necessary to assist the Senior Division Consultants. Senior Division Consultants will hereafter be responsible for the duties now being performed by the Division Consultants.

### Senior Divisional Surgical Consultants.

The Senior Divisional Surgical Consultant, under the Chief Surgical Consultant, A. E. F., will be expected to make at frequent intervals a complete survey of the professional instruction, surgical technique, and the methods of treatment in use in the Division, and he will render from time to time such reports and recommendations to the Chief Surgical Consultant, A. E. F., as will promote a free interchange of suggestions and the most effective co-ordination with the other professional services.

He will supervise the professional activities of all consultants, operating teams, and operating surgeons attached to his division, in a manner which will permit him to familiarize himself with the individual capabilities of the men, with a view to selection, based on observation of those likely to adapt themselves to modern military surgical team formations rather than individual work.

He will be responsible for the organization, efficiency, and distribution of surgical teams, and he will make such recommendations to the Chief Surgical Consultant, A. E. F., as will facilitate the formation of sufficient teams to meet the constantly increasing demands incident to the arrival in France of new formations.

The Senior Divisional Consultant will also coordinate the activities of the professional personnel in his division in a manner that will be conducive to high surgical standards, and elimination or reassignment to other duties of those who fall below the requirements. He will spare no effort to promote professional harmony and unity of treatment in the divisional formations.

### Senior Divisional Medical Consultants.

The Senior Divisional Medical Consultant will, by frequent inspections, satisfy himself that various classes of patients suffering from medical disabilities are receiving the best and most advanced treatment possible. He will report from time to time to the Chief Medical Consultant, A. E. F., the results of his inspections, and make suggestions looking toward the perfection of the Medical Service of the A. E. F.

### Divisional Surgical Consultants.

The Divisional Surgical Consultant will, under the Senior Divisional Surgical Consultant, supervise the immediate surgical activities of operating teams within his division. During mobile or semi-mobile warfare, when established evacuation hospitals are absent, the operative work, in formations for non-transportable cases, will be handled, when practicable, by surgical teams functioning under the supervision of the Senior Divisional Surgical Consultant, or his assistant.

### Divisional Medical Consultants.

Divisional Medical Consultants will supervise the immediate medical activities in the Division to which they may be assigned.

### Relations of the Divisional Surgeon to Senior Divisional Surgical Consultants and Consultants Functioning Divisions.

The many details of organization and administration which will devolve upon the Division Surgeon, in the care of sick and wounded and their evacuation, will so tax his time and ability that it is not believed that the supervision of the technical surgical work, which at times must be done in division formations, should be added to his already serious responsibilities; therefore, the direction and supervision of the purely operative side of the work done in divisional formations is placed upon the Senior Divisional Surgical Consultant, or his assistants .

The Division Surgeon will supply the necessary hospital facilities, supplies, and personnel other than those forming teams. He will spare no effort in technical cooperation which may promote harmony of action between the professional services with the fighting forces, from the front to the rear.

### Division Specialists.

One Orthopedic Surgeon, one Urologist, and one Neuro-psychiatrist will be appointed from the division sanitary personnel, and, under the direction of the divisional Chief Surgeon, they will perform the duties pertaining to their several specialities, in addition to the other duties of medical officers which may be required of them by the exigencies of the service.

### Consultants for Base and Hospital Centers.

Upon the recommendation of the Chief Surgical and Medical Consultants, A. E. F., there will be appointed for Base Hospital Groups such consultants as may be necessary from time to time. These consultants will at all times be within reach of the Base Hospital Group to which they are attached.

The organization of Base and General Hospitals and other hospitals, as far as practicable, will be made on the basis of three services : Surgical, Medical, and Laboratory, each composed of sections coordinated through a Chief of Service designated by the Commanding Officer, who may be selected from any section, ability and experience being the determining factors. In detail, the professional services of hospitals are divided according to the following outline :

### ORGANIZATION OF BASE AND GENERAL HOSPITALS

### Surgical Services

*Chief of Services :*

1st Section. — General Surgery. { General. Chest. Abdomen. Fractures.

2nd Section. — Orthopedic Surgery.

3rd Section. — Urology.

4th Section. — Head Surgery . . { Brain (also Neurology.)<br>Ear, Nose and Throat.<br>Eye.<br>Oral (Face and Mouth).

5th Section. — Roentgenology.
6th Section. — Dentistry.

### Medical Services

*Chief of Services :*

1st Section. — General Medicine.
2nd Section. — Neurology.
3rd Section. — Psychiatry.

### Laboratory Services

*Chief of Services :*

1st Section. — Pathology.
2nd Section. — Bacteriology and Serology.

### A. E. F. — DIRECTOR PROFESSIONAL SERVICES

Chief consultant, Surgical Service
Chief consultant, Medical Service

### ARMY

*Senior Consultant, Surgery, A. E. F.*

1. General Surgery.
1. Orthopedic Surgery.
1. Urology and Dermatology.
1. Eye.
1. Ear, Nose and Throat.
1. Neurological Surgery.
1. Maxillo-facial Surgery.
1. Roentgenology.
1. Research.
1. Formations, equivalent to an Army Corps.
4. Consultants (Assistants to division senior consultants).

*Senior Consultant, Medicine, A. E. F.*

1. General Medicine.
1. Neuro-psychiatry.
1. Formations, equivalent to an Army Corps.
2. Consultants (Assistants to division senior consultants).
(Others as required).

### ARMY CORPS

DIVISION

SPECIALISTS. — EACH TACTICAL DIVISION
(A Part of Division Sanitary Personnel, Tables of Organization).

*Surgery :*

1. Orthopedic Surgery.

1. Urology.

*Medicine :*

1. Neuro-psychiatrist.

### HOSPITAL CENTERS

*Consultants, Medicine :*

Each Hospital Center, S. O. S. :

1. General Medicine.

1. Neuro-psychiatric.

(Others as required).

*Consultants, Surgery :*

Each Hospital Center, S. O. S.

1. General Surgery.

1. Orthopedic Surgery.

1. Urology and Dermatology.

1. Eye.

1. Neurological Surgery.

1. Ear, Nose, and Throat.

1. Maxillo-facial Surgery.

1. Roentgenology.

### S. O. S. – SPECIALISTS. — EACH BASE HOSPITAL

(Part of Unit Personnel).

*Surgery* (as needed) :

General Surgery.
Orthopedic Surgery.
Urology and Dermatology.
Neurological Surgery.
Eye.
Ear, Nose, and Throat.
Roentgenology.
Maxillo-facial Surgery.

*Medicine* (as needed

General Medicine.

Psychiatry.

(Others as required.)

---

### EXTRACTS FROM WEEKLY BULLETIN OF DISEASE

### How to handle Diphtheria Carriers and Contacts When a Division Moves.

At present, when a division leaves a training area the camp hospitals are evacuated, carriers (whether of diphtheria or meningitis) held until freed from the infecting organism, must then be evacuated to a Base Hospital. The contacts of infectious diseases held for observation during the incubation period are taken along by the division with very imperfect provision for segregation. This has resulted in the development of a considerable number of secondary cases of various diseases and the spread of infection to previously uninfected units within a division. If the contacts, like the carriers, were sent to Base Hospitals they would then under existing conditions be sent from there to the replacement division of the corps and redistributed perhaps to some other division or unit of the corps.

It has been suggested that when a division leaves its training area a medical officer and perhaps a cook be left behind in the camp hospital with supplies sufficient for two weeks, and that contacts of recent infectious diseases, whose period of incubation is not yet over, be left under command of this medical officer for the remainder of that period. This would never be longer than two weeks, at the end of which time the final evacuation of the camp hospital could be completed and the contacts forwarded to their division in the line.

Several of the Division Surgeons have expressed their approval of this suggestion and, since there is a permanent personnel provided for camp hospitals, it rests with the Division Surgeon to use this method if he wishes to.

The practice in one division has been : " To appoint a surgeon and a small sanitary detachment for service with this detachment, and to leave behind an ambulance or two and the necessary medical equipment to care for the men of the detachment. It seems easier to assign the carriers and contacts, with necessary instructions for isolation, to the detachment left behind, rather than to leave a medical officer, a cook, and supplies in the camp hospital and allow them to fall into the hands of the S. O. S. ".

This procedure also would accomplish the desired results.

From another Division Surgeon comes the comment that, " As contagious contacts are not on the sick report, it would be difficult to have them placed under the command of a medical officer. It is suggested that when a division leaves its area, the contacts be segregated, left behind, and billeted in one locality, care being taken to arrange the billets so that contacts of only one disease are billeted in the same place. An officer of the line should be in command. The necessary cooks and cooking utensils should be provided. A medical officer should also be left with the men for the necessary examinations and medical attendance. Men in this group who develop the disease for which they are segregated should be evacuated to the nearest contagious disease hospital. "

This suggestion certainly meets the need in a simple way and perhaps with advantages over either of the previous plans.

Any method whereby the contacts could be held in the training area until the incubation time has passed and then be forwarded directly to their Division instead of going back to a base hospital and then through the replacement division, would, in our opinion, be satisfactory. There is not only delay in sending these contacts back to replacement divisions but there is danger inasmuch as they come in contact with a new group of men and in this way may act as carriers of disease.

### Special Notes on Diphtheria.

In two divisions diphtheria (in the United States, during transportation overseas; in France, in the billeting areas) has been present in such numbers and so continuously as to demand special assistance and attention from the Central Medical Laboratory.

The detection of all diphtheria carriers by culture of noses and throats of entire regiments is impracticable except at the cost of such delay as would neutralize any possible benefits to be expected. When diphtheria is confined to a unit no larger than a battalion, the search for carriers and susceptibles by culture and Schick test may properly cover all members of the command. Such methods, coupled with special attention to the kitchen personnel, the sanitation of billets, ventilation of sleeping quarters, and thorough washing of mess-kits have been successful in bringing epidemics to an end promptly.

When diphtheria appears distributed throughout an entire division, as, for instance, in 56 separate companies of organization in the ... Division, the problem is one of sanitation more than one which can be handled by laboratory methods exclusively. Under these conditions, the following steps are advised : Select for culture and Schick testing all known contacts of each case (sometimes as many as 30-40 may be necessary), i. e., bunk mates and those in any way intimately associated with the patient, in living, eating, working or social relations. Culture kitchen personnel and cooks of the patient's mess. Provide temporarily as much increased floor space per capita for the command as is practicable, resorting to shelter tents if necessary (in woods, when available, in advanced areas where enemy aviation makes tentage undesirable). Wet sweeping and thorough cleanliness of billets. Daily and thorough airing and sunning of bedding. Vigorous police control and discipline for spitting in or about billets, kitchens, and mess huts or sheds. Daily inspection of whole command for early detection of inflamed throats. Special care should be taken to provide *boiling water for washing mess-kits, under the supervision of a non-commissioned officer*. The last 50 or 60 men usually find a luke-warm, turbid fluid to rinse their dishes in. If fuel is lacking to provide *hot water* a generous allowance of warm soapy water must be provided and the dishes rinsed in a mild antiseptic solution.

### Regarding Circular 13.

Reports of "suspects" are often received during localized epidemics of diphtheria, scarlet fever, and meningitis, and these are but rarely followed by confirmation of diagnosis. It is important that, if a " suspect " is later found to be a true case of the disease in question, the case be reported on the day of diagnosis just as if it were a new admission. Only in this way can an accurate record of the number and distribution of cases of the important communicable diseases be kept for the Chief Surgeon.

### Hospital Commanding Officers to the Rescue.

The new method of reporting sick and wounded has been in operation since June 15th. It has satisfied every expectation. The office of the Chief Surgeon is equipped with the most modern methods for making use of the information which is received, and it now rests with the hospitals and the medical organizations in the field to take every precaution in making and forwarding reports so that this information can be properly used for the general benefit. The accuracy and promptness, with which the statistical tabulations from Form 22, Daily Report of Casualties and Changes, can be made depends directly upon the pains taken by be Commanding Officers of medical organizations to transmit the information correctly and regularly. The rapid transmission of these reports is all important and the established courier service should be utilized wherever possible.

### Effort Syndrome an Acceptable Term.

Since the abbreviation D. A. H., disordered action of the heart, and the three inclusions under this heading in the official nomenclature of the new Sick and Wounded Report (bradycardia, tachycardia, and arythmia) do not correctly express present day clinical facts, the term *effort syndrome* will be accepted as a diagnosis on the Field Medical Card and on daily hospital reports for the condition to which the term D. A. H., has been generally but incorrectly applied.

The condition was well known to military surgeons in the United States during the Civil War as the " irritable heart of soldiers ". This condition is a reaction to the stress, fatigue, and emotions incident to active warfare. Many cases occur also after infections such as trench fever and during convalescence after gassing. When suffering from this condition, the characteristic diagnostic points of the *effort syndrome* are complaint of pain in the chest, headache, giddiness, and breathlessness, all of which symptoms are aggravated by any inconsiderable effort. The heart rarely shows extreme slowing or quickening of the rate or irregularity in its rhythm.

The treatment consists in graded exercises, not in prolonged rest in bed, in encouragement as to early and complete recovery, not in calling attention to cardiac symptoms. The proper place for the treatment of these cases is in the convalescent camps now being established.

Hume in the *Lancet* (April 13, '18. p. 529), in reporting upon 5000 soldiers sent to the base with diagnosis of V. D. H. and D. A. H., says that 8.3 o/o were found to be suffering from easily recognizable diseases, in no way circulatory in origin. Of the other 91.7 o/o of the cases only 55 were cases of organic heart disease. The remaining cases complained chiefly of breathlessness, pain in the chest, palpitation, giddiness and other less easily definable symptoms.

" No matter how the condition may be produced, the rationale of treatment is the same in all cases. The most important factor in all cases is the abolition from the patient's mind of the idea that he has a diseased heart. Good food, undisturbed sleep and outdoor exercise, regulated and under discipline, are the sole factors necessary for the improvement of all types. By this method 50 to 60 o/o can be sent back to their original work after four to five weeks. The remaining 40 to 50 o/o are unable by reason of poor physique, actual disease, or age, to undertake every kind of work and hardship. Apart from those who suffer from actual heart disease or some organic disease of other systems, nearly all are fit for some service in France ".

REFERENCES TO CURRENT MEDICAL LITERATURE : INFECTIOUS JAUNDICE.

The problem of acute infectious jaundice in the United States with summary of experience among the troops of the nations at war in Europe is dealt with in an excellent article by M. H. Neill, Past Asst. Surgeon, U.S. Public Health Service in the Public Health Reports, Vol. 33, No, 19 of May 10, 1918. A full bibliography is appended. This publication can be obtained through the American Red Cross Medical Library, 12, place Vendôme, Paris.

### COMMANDING OFFICERS OF BASE HOSPITALS PLEASE NOTE

### TESTS FOR PHYSICAL DISABILITY.

Pending the establishment of convalescent camps, in order to prevent the return of men to duty before they are fit, patients no longer needing ward treatment should be segregated in convalescent wards in Base Hospitals for physical training under medical direction. Tests of fitness should precede discharge from hospital to duty. Functional test, even more than physical examinations, are needed to disclose latent circulatory and respiratory defects.

The following note from the President of a Disability Board puts the problem clearly :

" There are appearing before this board a class of cases from the hospitals whose disability has become apparent after discharge from the hospitals, which disability, it appears, is due to the impoverished physical strength of soldiers caused by hospitalization. Recovery from wounds and disease is complete and classification by boards at the hospitals as class " A " is correct when these only are considered; but travel, with its incident marching and other work, reveals a depleted physical condition, and the soldier's unfitness for active service or intensive training. That the soldier's convalescence is retarded and morale impaired by exertion not commensurate with his strength, we believe is possible, and we are of the opinion that the return of the soldier to active service through graduated training based on the individual requirement of the soldier will result in a saving of time and will prevent rehospitalization.

A second class consists of apparently convalescent gassed cases. These cases state they were well on discharge from hospitals, but as a result of the exertion necessitated by travel, shortness of breath has developed. Examination of the chest reveals emphysema accompanied with a tachycardia. It is the opinion of the board that a physical examination of the chest alone will not reveal the soldier's true physical condition and that classification on the chest findings is not entirely reliable. It is believed that classification in these apparently convalescent cases can be made more accurately by examination after carefully supervised exercise. "

### Prompt Evacuation of Class " D " Patents Imperative.

On account of the recent rapid increase and the contemplated large additions to our forces in France, it is necessary to exercise the greatest economy in the use of bed space in hospitals. Patients should be returned to duty as soon as their condition warrants such action.

Patients clearly falling under Class " D " should not be held for prolonged observation and study no matter how much professional interest they excite. Prompt action in the disposition of all Class " D " men is not only in the interest of the patients themselves, but will save hospital space and permit a steady flow of evacuables to home ports. Prolonged hospitalization in the Zone of Operations is to be avoided, since the authorized percentage of bed space in the A. E. F. contemplates final reconstruction and salvage of disabled men in the United States.

*Delay in autopsies.* The average time period between the death of individuals and the performance of autopsies in the A. E. F. is now 98 hours. In view of the rapid decomposition of dead bodies, particularly when refrigeration is impossible, it is desirable to reduce this delay as far as practicable. Organs and tissues are particularly valuable to the pathologists when they can be inspected and placed in fixation before cellular death has occurred. For fine cell studies, tissues should be fixed as soon after the circulation has ceased as possible.

### The Epidemic of Influenza.

This disease which was mentioned previously as " Three Day Fever " is now known to be due to the true Pfeiffer bacillus, although evidently of a much milder strain than the type which prevailed in the pandemic of 1889. This epidemic though of remarkable mildness in the two months from April 15 to June 15, has within the past four weeks shown certain characteristics which indicate the kind of increasing virulence with which bacteriologists are familiar in the case of strains of pneumococcus passed rapidly through a succession of susceptible animals such as the rabbit. Onset with temperature of 104, projectile vomiting, severe headache, Kernig's sign and high tension spinal fluid, flowing freely up to 100 cc. has not been uncommon and many cases have been mistaken for, and some have been treated as, meningitis with antimeningococcus serum. No harm, apparently, has come from the use of the treatment and symptomatic relief has often followed the withdrawal of the excess fluid, but confusion is likely to arise when the fluid clear on the first tap becomes turbid with leucocytes from the reaction following the introduction of the antimeningococcus serum. If the meningococcus is not found on smear or culture it is well to be satisfied with withdrawal of fluid and not to give serum. Pneumonias have been more common sequellae in July than in April.

Prompt hospitalization effectively guards against serious sequellae and is still the best treatment to be offered. Relief from headache by aspirin, abundant use of water internally, gentle laxative, and, when the temperature has fallen, a nourishing diet, have seemed to be the approved treatment. The same warning should be given now as always in treating influenza, i. e., warn against premature return to hard work. The epidemic is about at an end so far as

A. E. F. troops are concerned and has been throughout of a benign type, though causing considerable non-effectiveness.

### Urticaria and Acidosis.

These affections have been reported in considerable numbers and the following quotations from a divisional sanitary report bring up important questions of dietary for consideration. " The food has lacked the proper balance and the demonstrable result which has followed is, that in personal examination of about five hundred men, taken from different organizations, there is a wide-spread existence of urticaria, attributable, in part at least, to the use of native acid wines. A considerable amount of native wine is consumed by men of this command. The excessive use of this wine is injurious to American soldiers. Our soldiers have been accustomed to the use of sweetened soda waters and other non-alcoholic drinks, which at various camps in the United States were used in large quantities. Our soldiers make use of French wines to a large extent because it is the only beverage which can be purchased locally. When consumed in large quantities it is injurious, not only on account of the alcohol, but also on account of the acid which it contains. Instances of diarrhea have been manifest, but of short duration. Upon each occasion, investigation has revealed the source of the difficulty, and upon correction of the cause, a prompt return of good health ensued. The causes have been largely due to moldy bread and the ingestion of insufficiently cooled meat.

It is evident that the existing diet is exceedingly deficient in its sugar component. The use of syrup of fruit extractives, liberally supplied with sugar and water, would have beneficial results by reason of the several following advantages : *a*) To supply deficiency in sugar. *b*) Cause additional amount of water to be ingested. *c*) Replace native wines, thereby lessening the prevailing intestinal intoxication which has produced urticaria, and decrease the pyodermic hazard. *d*) Promote efficiency by reducing the amount of alcoholic intoxication.

The matter of cooking and addition of fresh vegetables to the diet of the men is an important one. It is possible in this district to buy a number of different kinds of fresh vegetables such as spinach, asparagus, fresh onions, cauliflower, cabbage, and rhubarb. The issue of rations in kind which are shipped from the Central Supply Depot does not provide for these articles. Fresh vegetables cannot well be purchased by the Central Supply Depot, and, if supplied from there, would lose much of their value on account of delay in shipment. French troops in this area make use of fresh vegetables which are procured from local farmers, and they are thereby able to give their men better diet than that which is supplied to American soldiers. The allowance of the saving privilege for purchase of fresh vegetables is strongly recommended."

### The Medical Officers' Responsibility.

In a summary of studies upon the disabilities in the sphere of psychopathology, the following statement of the medical officers' responsibility was made in a British publication.

"If we survey the present day field of psychopathology, I think the most comforting piece of scientific knowledge the war has brought to us is that the resistance which the physically sound person has against the injurious circumstances of war is extraordinarily great; that the popularly held opinion concerning the damaging influence of over-exertion and emotion upon an ordinarily sound, healthy mental constitution is not true. This information must be reflected in every consideration of capacity for service. Every pension wrongly granted after discharge from military service damages our social body in a twofold manner. The wrong granting of pension is the lesser of these, the hindering suggestion of incapacity for work which we give to the man in his life is far greater. Every workman will be necessary in the coming times of peace, and we doctors therefore carry on our shoulders a very great part of the responsibility for the return of the discharged soldiers to work; on the grounds of false humanity we have no right, at the cost of the community at large, to give to these men too freely."

### Fly Prevention.

The tangle-foot wire is used on a large scale and with excellent results in the B. E. F., as many as 10,000 such wires being distributed each week by the sanitary section in charge of a collection of hospitals and convalescent camps at one Base.

Heat 4 pints of castor oil in an open pan. Stir in 9-1/2 lbs., crushed resin. Continue heating and stirring for about one hour until the resin is dissolved and the mixture is of paint-like consistency.

Apply to wires with brush or by dipping while mixture is still hot.

When wires become covered with flies, collect, place in fire, burn off flies, wipe dry with rag or newspaper, re-coat and re-hang.

In the first ten days of July, 12 cases of amebic dysentery were reported. In April, May and June there were three cases each month. The increase of dysentery with hot weather reminds us that in spite of the relatively small amount of fly breeding in France as compared with the United States, we must put human

A. Method of hanging wires. B. Detail of wire. — Hay bale wire 2 ft. - 0 in. long, C. Cresol can carrier. Rope handles.

FLY-PROOF LATRINE

If concrete floor is built place nailing blocks in floor.
Minimum distance between seat holes is 1 ft. - 0 in.

excreta out of reach of flies, and keep flies away from food. Careful carpentry will make a latrine fly-proof. Incineration is the best method of feces disposal.

### Beware of Acid Solutions of Arsenobenzol.

At a Base Port it was found that five syphilitics had been treated with *arsenobenzol* in acid solution and in high concentration, the surgeon supposing that he was using novarsenobenzol. This mistake has also occurred at at least one base hospital. The arsenobenzol is much more readily soluble in water than the salvarsan which we have in the U.S. This encourages such a mistake. The five cases above-mentioned were desperately sick and ended by having pneumonia with bloody expectoration, but all recovered. Such a mistake can obviously not be made often without an occasional fatal result. Arsenobenzol corresponds to salvarsan and requires dilution and alkalinization. Novarsenobenzol, which corresponds to neosalvarsan does not require alkalinization. If the arsenobenzol brand of arsphenamin made in the United States is used as directed, i.e., accurately diluted and alkalinized, it will produce no untoward results.

A report in the *Journal of the American Medical Association* of May 18, 1918, p. 1458, by Capt. Meddis and Lt. Stirling, M.R.C., upon 1104 injections of arsenobenzol at Camp Zachary Taylor at Louisville, Ky., presents the following conclusions: "1. The arsenobenzol brand of arsphenamin made in this country (U.S.) is in our experience non-toxic, and as efficient therapeutically as the original Ehrlich preparation. 2. It may be used in concentrated solution with no ill effects. 3. Epinephrin, given in a 1 : 1,000 solution ten minutes before the injection, will control reaction. 4. The only reaction noted in this series of cases was slight headache; in some cases, diarrhea and slight malaise were noted. 5. In phagedenic chancroids, "arsenobenzol" has a very beneficial effect, and is recommended where the healing is slow and response to other treatment is poor."

### When Not to Operate in Tuberculosis of the Kidney.

A number of cases of tuberculosis of the kidney have been diagnosed and operated upon in the A.E.F. Unfortunately the wound in the loin often breaks down and takes from three months to a year to heal, during the greater portion of which time it is improper to move the patient from the hospital. This complication is so common and need for operation in these cases so little pressing that surgeons are urged not to perform it on this side of the water, but to send the patients home with a diagnosis for operation.

### Death from Infectious Jaundice.

Private...... Engrs. 28, white, nine months in service, admitted to a Base Hospital June 7th, diagnosed "cholecystitis acute, catarrhal and jaundice, acute, infectious," and died June 26th. Jaundice 21 days, no nausea, no enlargement of liver. At first no abdominal tenderness, later in upper half, pulse 60. Important findings at necropsy were, — " Bronzed skin, undernourished; peritoneal surfaces smooth, no increase of fluid, loose adhesions about gall bladder. Brown pigmentation of heart. Edema of lower lobes of lung. Spleen normal. Liver: gall bladder small, walls thickened, contains thick bile-stained mucus. Mucosa pale and normal. Bile passages patent and normal. Liver small, dark red, flabby, leathery, lobules distinct, small areas of yellow. Entire organ jaundiced. Intestines contain bile — colored feces. Kidneys: large, cortex thick glomeruli red, organ jaundiced. Anatomical diagnosis: Fibrosis, fatty degeneration and necrosis of liver, chronic cholecystitis of Kidney. Bacteriological: no growth from heart or spleen. Probable cause of death: infectious jaundice."

### Increase in Strangulated Hernias in Germany.

An unexpected result of the low-fat or diet in Germany has been a great increase in the number of cases of strangulated inguinal and internal intestinal hernias, requiring immediate surgical treatment. To the disappearance of adipose layers around the intestines, lack of peritoneal support for bowels, and increase of cellulose in the food which stimulates intestines to greater activity are attributed the increase.

### Mange a Human Affection During This War.

(Extract from "Medical Supplement" Feb., 1918) — *Horse Mange*: — In peace horse mange was not common in the Central Empires, but during the war it has been imported from Poland and Serbia and has spread to man and even from man to man; Pick, who examined a number of cases in unfavorable circumstances, thus contracted it. He has never seen dermatokoptes or dermatophagus mange but always the sarcoptes form. The incubation period is short, two or three days, and then groups of small pale nodules the size of a pin's head appear on the back, trunk, flexor surfaces of the upper extremities, groins and thighs, the distribution being ascribed by Reif to hair and scales falling down inside the collar. The wrist, hands, penis, face and scalp escape. As in scabies, itching, when the patient gets warm in bed, is troublesome; but, unlike scabies, there is no associated impetigo or eczema. Burrows are not seen, probably because the parasite is more superficially placed than the Acarus scabiei in the skin. Spontaneous cure occurs in one to three weeks. Prophylaxis consists in washing with carbolic soap, or rubbing on petroleum after working in the stables.

As a cure Weidner speaks highly of petroleum (ʒ i), rubbed on the skin until it is dry, once daily; mild cases are cured by a single application, severe ones after two to four. The clothes and bedding should be disinfected.

### Eczema Marginatum.

This familiar disease due to Epidermophyton inguinale and better called tinea marginata has become extremely common in the Central Empires, and according to Galewsky is often caught in baths and latrines. Unless carefully disinfected the thermometers in hospitals may spread it. Schillenberg recommends artificial sun baths as better than ordinary iodin treatment. In extensive or chronic cases Muller observed excellent results from exposures to a quartz lamp at a distance of 30 to 65 centimeters for 5 to 30 minutes.

### Trachoma Among Chinese Laborers.

Where Chinese laborers are used, precautions must be taken to avoid the introduction and spread of trachoma among the American Expeditionary Forces. Trachoma is common among the Chinese, and some of the large Chinese labor camps of the B. E. F. provide for three groups, those who have active discharging and infective conjunctival lesions, those who have the disease in the quiescent and non-infectious stage, but in whom irritation or inflammation of the conjunctiva from other cause may develop a recrudescence, and those with normal conjunctivae. An acute outbreak of conjunctivitis was recently reported among the Chinese working at one of the camp hospitals.

### Rabies.

Again a warning has been issued by the French health authorities as to the dangers of rabies, a marked increase in the number of rabid dogs having occurred in March, April, and May. Rabies is common throughout France. One death from Rabies in the A. E. F. has been already reported. Report of dog bites should be made to medical officers so that suitable treatment of the wound may be given. The dog should be held for observation under authority of the local French Veterinary service. If antirabic treatment is considered necessary the Central Medical Department Laboratory at Dijon should be notified promptly.

### Anthrax.

There have been 13 cases of anthrax in the A. E. F. since March 30th. Of these all but two occurred in men who had just arrived on transports, or developed during the voyage. Of the other two one developed the lesion at the site of a cut made while shaving. In several lots of shaving brushes collected from among arriving troops the bacillus anthracis has been found by bacteriologists in England and in France. Cases have recently been reported in the United States from several of the cantonments. A special study of the industry and the source of the infected bristles (imitation badger hair) which are supposed to be horse hair from endemic centers of anthrax in Siberia, Manchuria, or Argentine is now being made by the United States Public Health Service. Any information as to place and time of purchase and make of shaving brushes which have been used by patients found suffering from anthrax will be helpful in tracing and discontinuing the source of the infection. Appropriate and prompt surgical treatment of localized lesions, supplemented when indicated by specific antiserum (obtained on request from the Pasteur Institute in Paris) will save life in cases correctly diagnosed. The following errors of diagnosis have been reported.

1. Anthrax of neck : primary diagnosis, cellulitis of neck following vaccination.
2. Anthrax of neck : primary diagnosis unilateral mumps.
3. Anthrax septicemia : primary diagnosis, meningitis.
4. Staphylococcus abscess of neck : primary diagnosis, anthrax.

Suspected anthrax has been reported in the following organizations.

308 Field Artillery, Battery A.
309 Field Artillerie, Cos. B. & D.
303 Trench Mortar Battery.
403 Ammunition Train, Hq. Co. Cos. C. & D.
115 Field Artillery, San. Detach. Bty. E.
303 Supply Train, Co. E.
303 Motor Supply.

*Differential Diagnoses* of interest in the past week.

1. Reported dysentery; correct diagnosis, syphilis.
2. Reported typhoid : corrected diagnosis, bronchitis.
3. Reported suspected meningitis (50 cases), corrected diagnosis epidemic " Three day fever ".
4. Reported suspected typhus or typhoid : correct diagnosis miliary tuberculosis.

*Le Gérant :* O. Porée. Paris — Imp. Lahure, 9, rue de Fleurus.

## Figures and Tables

**Figure f1:**
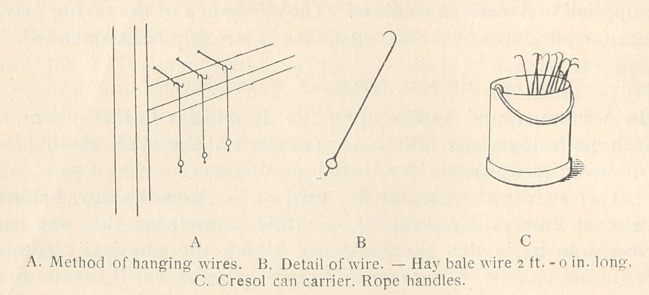


**Figure f2:**